# Estrogen Receptor α Functions in the Regulation of Motivation and Spatial Cognition in Young Male Rats

**DOI:** 10.1371/journal.pone.0079303

**Published:** 2013-11-13

**Authors:** Katrin Meyer, Volker Korz

**Affiliations:** 1 Leibniz Institute for Neurobiology, Magdeburg, Germany; 2 Institute for Biology, Otto-von-Guericke University, Magdeburg, Germany; 3 Center for Behavioral Neuroscience, Magdeburg, Germany; Tokai University, Japan

## Abstract

Estrogenic functions in regulating behavioral states such as motivation, mood, anxiety, and cognition are relatively well documented in female humans and animals. In males, however, although the entire enzymatic machinery for producing estradiol and the corresponding receptors are present, estrogenic functions have been largely neglected. Therefore, and as a follow-up study to previous research, we sub-chronically applied a specific estrogen receptor α (ERα) antagonist in young male rats before and during a spatial learning task (holeboard). The male rats showed a dose-dependent increase in motivational, but not cognitive, behavior. The expression of hippocampal steroid receptor genes, such as glucocorticoid (GR), mineralocorticoid (MR), androgen (AR), and the estrogen receptor ERα but not ERβ was dose-dependently reduced. The expression of the aromatase but not the brain-derived neurotrophic factor (BDNF) encoding gene was also suppressed. Reduced gene expression and increased behavioral performance converged at an antagonist concentration of 7.4 µmol. The hippocampal and blood serum hormone levels (corticosterone, testosterone, and 17β-estradiol) did not differ between the experimental groups and controls. We conclude that steroid receptors (and BDNF) act in a concerted, network-like manner to affect behavior and mutual gene expression. Therefore, the isolated view on single receptor types is probably insufficient to explain steroid effects on behavior. The steroid network may keep motivation in homeostasis by supporting and constraining the behavioral expression of motivation.

## Introduction

In recent years, estrogen receptors have increasingly been identified as involved in modulating motivation and cognition in female human development, postmenopausal mood disorders, and corresponding animal models [1], [2], [3]. The effects in male subjects, however, have been largely neglected, although the entire enzymatic machinery for locally producing estrogens as well as both estrogen receptors (ERα and ERβ) are present in male brains. Moreover, there is evidence that cognitive deficits can be rescued by estrogens [4]. Most studies focused on sexual and aggressive behavior [5], [6], [7]. The large body of evidence of estrogenic effects on neuronal plasticity, such as long-term potentiation, spine plasticity, and neurogenesis [8], [9], [10], [11], is contrasted by only a few studies on the effects of more general states such as motivation and mood and their outcome in behavioral performance. Aggression and modulations of the stress axis activity have been reported to be affected by estrogenic mechanisms in male mice and rats [12], [13], [14]. Aggression, therefore, may be stimulated by ERα and suppressed by ERβ activation in male rats [15]. Estrogenic effects in spatial learning have also been reported in both sexes [16], [17], [18], [19], [20], [21].

Hippocampal synthesis of estradiol in male rats is realized by the enzyme aromatase that converts testosterone into estradiol. Aromatase as well as synaptic and nuclear ERα have been identified in all subregions of the hippocampus and in the dentate gyrus. Estradiol can induce rapid upregulation of spine number and fast modulation of hippocampal synaptic plasticity [22]. Accordingly, rapid alternation and control of various behaviors in males, including learning, are controlled by brain-derived estrogens [23].

In a previous study [24], we found a positive correlation between hippocampal ERα gene expression and the behavioral performance of young post-pubertal male rats in a spatial holeboard paradigm. The motivational (i.e.,task readiness) component, which was extracted with principal component analysis from numerous behavioral elements, in particular was strongly correlated with the expression of ERα and weakly correlated with the testosterone binding AR, whereas ERβ, MR, and GR receptor gene expression was uncorrelated with components representing motivation, spatial cognition, and emotion. Therefore, in the present study, we applied the ERα-specific antagonist methyl-piperidino-pyrazole (MPP) in four dosages to reveal more specific functions of ERα activity in motivation and spatial cognition during our holeboard paradigm in male rats. Steroid receptors are assumed to act in concert and mutual interactions via heterodimerization [25] and other protein-protein interactions [26], [27]. Ligand-activated cytosolic ERα translocates into the nucleus where, similar to all steroid receptors, this receptor acts as a transcription factor, activating or repressing the expression of target genes including those of other steroid receptors. Thus, we measured the expression of hippocampal corticosterone binding receptors, the MR and GR genes, as well as the AR and both estradiol binding estrogen receptors. In addition to these slow genomic functions, membrane-bound steroid receptors can mediate fast non-genomic functions by activating different intracellular pathways [28]. Membrane receptors that are at least closely related to ERα and ERβ due to their ability to be activated by ERα- or ERβ-specific agonists have been identified [29]. Membrane-bound estrogen receptors are also involved in hippocampal-dependent object memory consolidation [30].

Therefore, while the MPP binds to cytosolic ER, we cannot rule out that membrane-bound receptors are blocked as well. The blood serum and hippocampal stress and sex hormone concentrations were also measured. In addition, aromatase and BDNF gene expression was measured. The latter was considered because rat hippocampal BDNF has been reported to interact with estrogens such that the administration of estradiol enhances hippocampal BDNF-mRNA [31], [32], by activation of extranuclear ER [33], whereas corticosterone [34] reduces BDNF-mRNA, and thus have opposite effects on BDNF expression and possibly on BDNF effects on learning and memory.

Based on our previous data, we hypothesized reducing the motivation of males treated with an ERα antagonist, whereas the spatial cognitive aspect should be unaffected. The latter hypothesis was confirmed, whereas MPP treatment resulted in increased motivation. Possible reasons for these results are discussed, and advanced hypotheses are outlined.

## Methods

### Ethics Statement

All experiments were performed in accordance with the European Communities Council Directive of 24th Nov. 1986 (86/609/EEC) and the German guidelines for the care and use of animals in laboratory research. The experimental protocols were approved by the ethics committee of Saxony-Anhalt. All efforts were made to reduce the number of rats used in this study and their suffering.

### Animal Keeping

Wistar rats from the institute’s breeding colony were weaned at post-natal day 21 and then housed in groups of 5 males in standard cages (59 cm×38 cm×25 cm). They were maintained under a 12 h: 12 h light regimen with lights on at 6∶00 a.m.; the ground was covered with commercial bedding material (wood spans, ssniff, Soest, Germany) and food pellets (ssniff,/M-H), and tap water was given *ad libitum*. After the implantation of a cannula when the rats were 7 weeks old, they were transferred to individual cages (40 cm×25 cm×18 cm).

### Surgery

Seven-week-old rats were anesthetized with Nembutal (40 mg/kg, intraperitoneal.). An intra-cerebroventricular (i.c.v.) cannula was stereotactically implanted in the lateral ventricle of the right hemisphere (coordinates: AP –0.8; L 1.5 from the bregma). The animals were allowed to recover from surgery for at least 3 days. After recovery, the animals were mildly food deprived for 4 days (receiving 5–6 g food per day, thus reaching about 80% of the initial body weight); food deprivation persisted during the training procedure. Body weights between groups were not statistically different at all time points:day −3 (three days before habituation; F_5,80_ = 1.44, p = 0.219), day 0 (habituation; F_5,80_ = 1.27, p = 0.287), day1(first training day; F_5,80_ = 1.87, p = 0.109),and day 2 (second training day; F_5,80_ = 1.52, p = 0.194).

### Behavioral Tests

#### Holeboard

The holeboard apparatus consisted of a black board (1 m×1 m) with 16 regularly arranged holes, 7.5 cm in diameter and 7 cm deep. The board was surrounded with Plexiglas walls 50 cm high. The walls were covered with white paper on the outside and marked with a different black cue at each side. Four out of 16 holes were baited in a fixed pattern with standard food pellets (dustless precision pellets, 45 mg, BioServ).

Path trajectories were recorded with the tracking system BiObserve Viewer software (Version 3.0.0.92), and behavioral parameters were measured. The head-dips of the animals were registered by photobeams at the middle of each hole. The photobeam signals were detected and counted by the holeboard-plugIn of the BiObserve Viewer software.

Thus, the time to find all pellets (the latency for rats that did not find all four pellets was scored as 120 s), the average velocity (given as the average speed in cm/s during a given trial), the number of pellets found, the hole dips/s, the total hole dips, the mean distance to the wall (the distance from the animal’s body to the nearest wall, given as the mean over all time points of a trial), the working memory errors (a rat revisits a hole that it already took bait from during a specific trial), and the reference memory errors (visiting an unbaited hole) were recorded. We calculated an index of performance for both types of memory. This calculation helps to divide the animals that made no errors, because they did not move at all on the holeboard from those that made no errors because they performed the task correctly. The index was calculated as total visits/(error+total visits). Thus, an index of 0 indicates no hole visit at all (0 errors because of 0 attempts were set to 0), 0.5 the number of hole visits equals the number of errors, and 1 indicates no error (four dips coincides with four pellets found).

Animals stayed in their home cages in the testing room during the entire experiment. Animals were transferred from the cage to the test arena for each training trial. A trial was automatically stopped after 2 min or when the animal had found all 4 pellets. All experimental animals were familiarized with the test set-up 1 day before training. Then they received spatial training on a fixed pattern of baited holes over 10 trials (day 1: five trials, day 2: four trials, and the retention trial on day 3), with a 15 min inter-trial interval [35]. Training started at 9∶00 am, and the retention trial (day 3) started 24 h after the last trial of day 2 (10∶00 am). The board was cleaned after each trial with 20% ethanol.

#### Open-field test and elevated plus maze test

Animals were tested for 5 min in each test twice: open field at 9∶00 a.m. followed by the elevated plus test 1 h later. This procedure was repeated 24 h later. Animals stayed in the test room overnight. For these tests, we used non-food-deprived animals. The arenas were cleaned after each trial with 20% ethanol.

#### Open-field test

The holeboard served also as an open-field arena, with the exception that the floor with the holes was covered with a black plastic plate. The arena was, via the tracking system, divided into different zones: four corner zones (25 cm×25 cm), four wall zones (25 cm×50 cm), and one center zone (50 cm×50 cm). The percentage of time spent in each zone, the mean velocity, and the track length were measured.

#### Elevated plus maze

The elevated plus maze consisted of two closed and two open arms of black plastic at a height of 80 cm above floor. The arms were 10 cm wide and 50 cm long. The closed arms were equipped with black walls (30 cm high). From a center arena (10 cm×10 cm), the animals started to explore the maze. The percentage of time spent in each arm, the mean velocity, and the track length were measured via the BiObserve Viewer software.

### Pharmacology

The ERα antagonist MPP binds to extranuclear receptors with very high specificity [36] and has been proved to antagonize estradiol-induced gene transactivation and -repression with no effect on these processes mediated through ERβ [37]. MPP, dissolved in water, was administered in four concentrations, 1.5 µmol (n = 18), 3.7 µmol (n = 13), 7.4 µmol (n = 18), and 12.6 µmol (n = 12), and applied over 7 consecutive days (starting 5 days before the experiments and ending at training day 2) via a Hamilton syringe (5 µl volume over 5 min). A flexible tube allowed the animals to move freely during administration. Two food-deprived, vehicle-treated groups, one trained (n = 13) and one untrained (n = 7), served as time-matched controls. The untrained control group remained in the testing room throughout the experiments. All animals in the various groups were killed euthanized at the same time point (15 min after the retention trial, i.e., between 10∶15 and 10∶30 a.m.).

Similar as in previous studies, we investigated the right hippocampus (genes, hormones) as well as the hormone levels in blood serum.

The rats used for the open-field and elevated plus maze tests were treated with 7.4 µmol MPP i.c.v. (the most effective dose in the holeboard task) or vehicle. In accordance with the holeboard procedure, daily injections started 5 days before the first test day and continued during the two test days.

### Tissue Sampling and Hormone Assaying

Animals were decapitated 15 min after the last trial, and trunk blood was collected in vials containing clot activator (REF 41.1500.005, Sarstedt; Germany). Tissue from the right hippocampus was rapidly dissected and frozen (−20°C) until measurements. Samples were homogenized (Biovortexer No. 1083; BioSpec products), diluted (Sample diluent; IBL Hamburg; REF KLZZ731) to reach a final volume of 25 µl/mg tissue weight, and centrifuged (10 min, 10000 rpm). The supernatant was stored at −20°C. For the hormone assays, we used the enzyme-linked immunosorbent assay (ELISA). Briefly, samples were thawed and diluted (brain samples 1∶3, 1∶2, and 1∶1, serum samples 1∶10, 1∶2, and 1∶5 for the testosterone, 17β-estradiol, and corticosterone assays, respectively). Samples and standards were applied in duplicate. OD values were measured at 450 nm in a micro-plate reader (Thermo Scientific MultiSkan FC ELISA Reader) and calculated via a standard four-parameter logistics plot. For the testosterone assay (Testosterone Saliva ELISA by IBL Hamburg; Germany), the limit of detection (LOD) was 2.0 pg/ml, and the intra-assay and inter-assay coefficients of variation were 8.2% and 5.5%, respectively. The estradiol assay (17beta-Estradiol Saliva ELISA by IBL Hamburg) had an LOD of 0.4 pg/ml. The intra-assay and inter-assay coefficients of variation were a maximum of 9.9% and 11.1%, respectively. For the corticosterone kit (Corticosterone ELISA by IBL Hamburg), the LOD was 1.631 nmol/L, and the intra-assay and inter-assay coefficients of variations were 2.77% and 6.14%, respectively. Randomly chosen subsets of the animals used in the behavioral experiments were analyzed.

### Quantitative Real-time RT-polymerase Chain Reaction

Tissue from the right hippocampus, added with an mRNA stabilizing agent (RNA later, Qiagen, Hilden; Germany), was stored at −80°C. For the analysis, mRNA was isolated (RNeasy Plus MiniKit, Qiagen) and transcribed to cDNA (high-capacity cDNA reverse Transcription Kit from Applied Biosystems (Carlsbad, CA, USA, now Life Technologies).

Gene expression analysis was performed with a StepOnePlus machine from Applied Biosystems. TaqMan Gene Expression assays with the classification “m” were used exclusively, whose primers (Life Technologies) span an exon junction and detect only genomic DNA, except PGK, where no assay with the classification “m” was available (AR/*AR*: NM_012502.1 (Rn00560747_m1); ERα/*ESR1*: NM_012689.1 (Rn01430443_m1); ERβ/*ESR2*: NM_012754.1 (Rn00562610_m1); GR/*NR3C1*: NM_012576.2 (Rn01405584_m1); MR/*NR3C2*: NM_037263.1 (Rn00565562_m1); BDNF/*BDNF*: BC_087634.1 (Rn01484928_m1); Aromatase/*Cyp19*: NM_017085.2 (Rn01422547_m1); PGK/*PGK*: NM_053291.3 (Rn00821429_g1); HPRT/*HPRT*: NM_012583.2 (Rn01527840_m1);TaqMan Universal PCR Master Mix). Assays were performed in triplicate. As endogenous control, we used rat GAPDH (Vic/MGB Probe), while HPRT and PGK served as reference genes. All reagents were from Applied Biosystems. Differences in relative gene expression were calculated with the comparative cycle threshold method [38]. The relative expression levels of estrogen receptor genes ERα and ERβ, AR, MR, and GR as well as the BDNF and aromatase encoding genes were measured. Subsets of the animals used in the behavioral experiments were analyzed because of material limitations (serum) or were chosen randomly (brain samples).

### Statistical Analyses

All statistical analyses were conducted with SPSS (V. 20). The distribution of all data was tested with the Shapiro-Wilk-test. Differences in gene expression data and hormone concentrations were analyzed with the univariate general linear model (GLM) with the treatment as a factor followed (if significant) by post-hoc tests (Tukey). Data that were not normally distributed (reference and working memory errors) were analyzed with the Kruskal-Wallis test for k-group comparisons followed (if significant) by the Mann-Whitney-U-test for pairwise comparisons. Behavioral data over the trials were tested with a linear mixed model with trials, learning phases (days), treatment, and phase×treatment interaction as fixed factors and trials and learning phases as repeated measure variables. The linear mixed model, in contrast to the general linear model, compares the phases with different numbers of trials and handles missing values. Behavior in the open-field and elevated plus maze tests was analyzed with the GLM for repeated measures. All tests were two-tailed, and the level of significance was set at p≤0.05.

## Results

### Behavior

**Figure 1:**
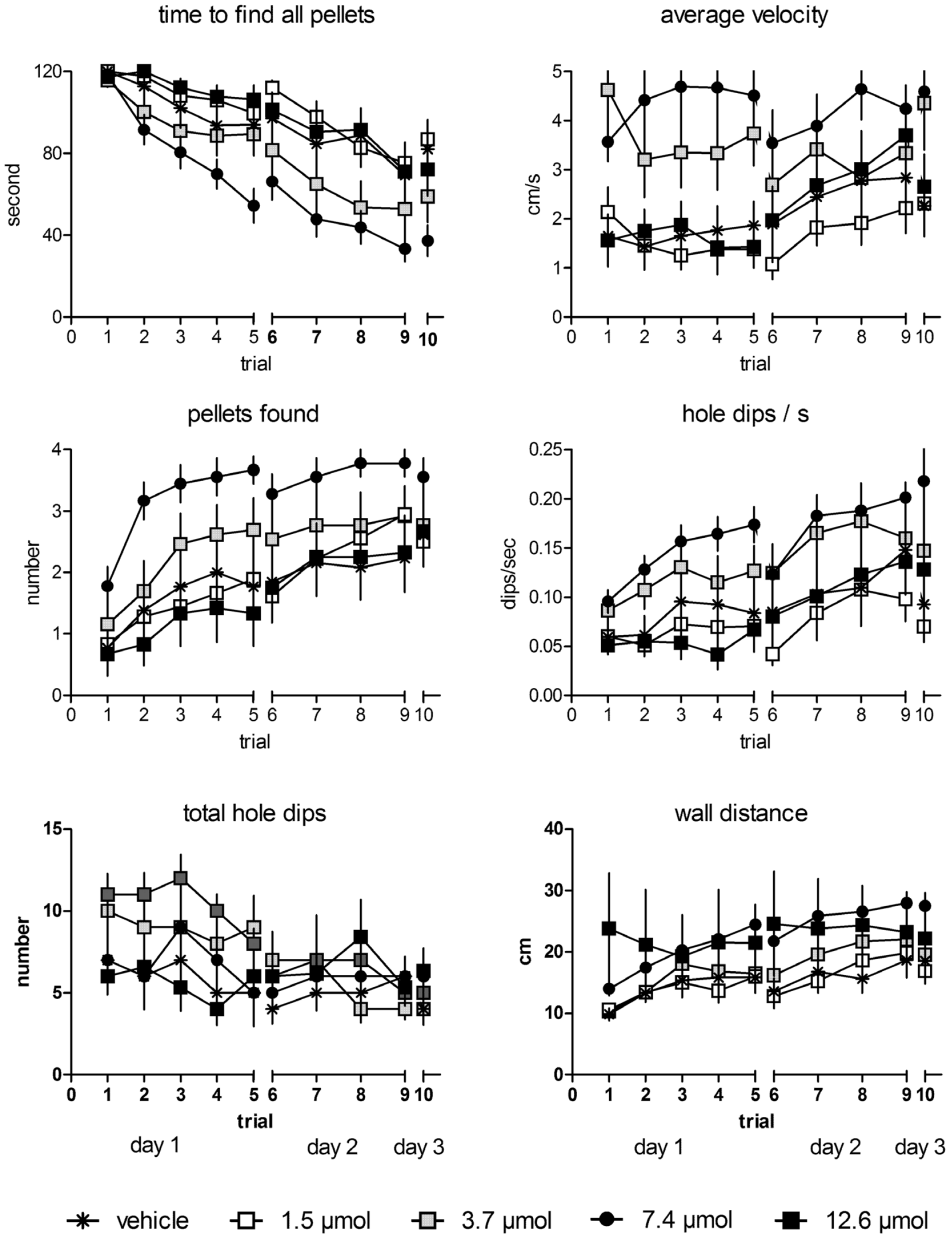
Treatment with MPP at a 3.7 and 7.4 µmol concentration decreased the time to find all pellets and increased the average velocity when compared to vehicle controls. Only animals treated with 7.4 µmol MPP found significantly more pellets within 2 min, showed increased numbers of hole dips/s and total hole dips as compared to vehicle treated rats and larger mean wall distances were shown in rats treated with 7.4 and 12.6 µmol MPP when compared to controls. Given are the arithmetic means and the s.e.m.

We analyzed six behavioral parameters: the latency to find all pellets, the average velocity, the number of pellets found, the average dips per second, the total number of hole dips and the mean distance of the rats to the wall (Figure 1). The outcome of the statistical analysis is summarized in Table 1. We found significant overall differences (test of fixed effects) for all parameters regarding the treatment, trial effects could be determined only in the time to find all pellets, the number of found pellets and the number of hole dips/s. A significant interaction of dose and learning phase appeared only for the time to find all pellets. The estimates of the fixed effects that animals treated with 3.7 µmol MPP showed significantly decreased slopes at acquisition phases 1 and 2 but not during retention, whereas the decrease in the group treated with 7.4 µmol MPP was significant in all phases. Pairwise comparisons of estimated marginal means revealed significantly faster times to find all pellets, higher velocity, more pellets found, more total hole dips and hole dips/s and a larger wall distance for the group treated with 7.4 µmol MPP (Video S1), faster times to find all pellets and higher velocity for the animals treated with 3.7 µmol MPP, and larger wall distances of rats treated with 12.6 µmol MPP compared to vehicle-treated rats (Video S2).

**Table 1:** Statistical analysis of differences in behavior.

behavior	tests of fixed effects (type III)				estimates of fixed effects				pairwise comparisons		
	effect	df	F	p	effect	df	T	p	pair	df	p
time to find all pellets	trial	7/124.007	21.99	**<0.000**	acquisition1×3.7 µmol	83.838	−2.134	**0.036**	1.5 µmol/vehicle	103.88	1.000
	dose	4/103.880	14.29	**<0.000**	acquisition2×3.7 µmol	277.495	−2.895	**0.004**	3.7 µmol/vehicle	103.88	**0.024**
	phase×dose	8/127.358	6.75	**<0.000**	acquisition1×7.4 µmol	83.838	−2.422	**0.018**	7.4 µmol/vehicle	103.88	**<0.000**
					acquisition2×7.4 µmol	277.495	−5.399	**0.000**	12.6 µmol/vehicle	103.88	1.000
					retention×7.4 µmol	69	−3.038	**0.003**			
average velocity	trial	7/147.923	1.15	0.338					1.5 µmol/vehicle	124.906	1.000
	dose	4/124.579	18.58	**<0.000**					3.7 µmol/vehicle	124.906	**0.001**
	phase×dose	8/142.628	1.62	0.123					7.4 µmol/vehicle	124.906	**<0.000**
									12.6 µmol/vehicle	124.906	1.000
pellets found	trial	7/137.952	7.37	**<0.000**					1.5 µmol/vehicle	132.579	1.000
	dose	4/132.579	12.96	**<0.000**					3.7 µmol/vehicle	132.579	0.331
	phase×dose	8/145.105	0.54	0.825					7.4 µmol/vehicle	132.579	**<0.000**
									12.6 µmol/vehicle	132.579	1.000
hole dips/s	trial	7/126.841	4.94	**<0.000**					1.5 µmol/vehicle	88.989	1.000
	dose	4/88.989	13.68	**<0.000**					3.7 µmol/vehicle	88.989	0.073
	phase×dose	8/115.733	0.95	0.482					7.4 µmol/vehicle	88.989	**<0.000**
									12.6 µmol/vehicle	88.989	1.000
total hole dips	trial	7/136.507	1.14	0.341					1.5 µmol/vehicle	206.706	0.562
	dose	4/206.706	8.01	**<0.000**					3.7 µmol/vehicle	206.706	0.915
	phase×dose	8/165.478	4.50	**<0.000**					7.4 µmol/vehicle	206.706	**0.005**
									12.6 µmol/vehicle	206.706	0.590
wall distance	trial	7/137.488	2.01	0.058					1.5 µmol/vehicle	199.164	1.000
	dose	4/199.164	12.98	**<0.000**					3.7 µmol/vehicle	199.164	0.981
	phase×dose	8/166.839	1.69	0.103					7.4 µmol/vehicle	199.164	**<0.000**
									12.6 µmol/vehicle	199.164	**<0.000**

Statistical analyses of differences in behavior. Linear mixed model with trial, learning phase (phase: acquisition1, acquisition2, retention), the MPP-dose and the learning phase×dose as factors and behavior as dependent variable. Trial, MPP-dose and learning phase×dose interaction as fixed effects. Trial and phase as repeated measures variables with diagonal covariance type. Estimates of fixed effects are given only for the interactions (controls as reference category) and only significant results. Main effects of estimated marginal means. Significant results are indicated in bold (p-values of pairwise comparisons of estimated marginal means are Bonferroni-corrected).

**Figure 2:**
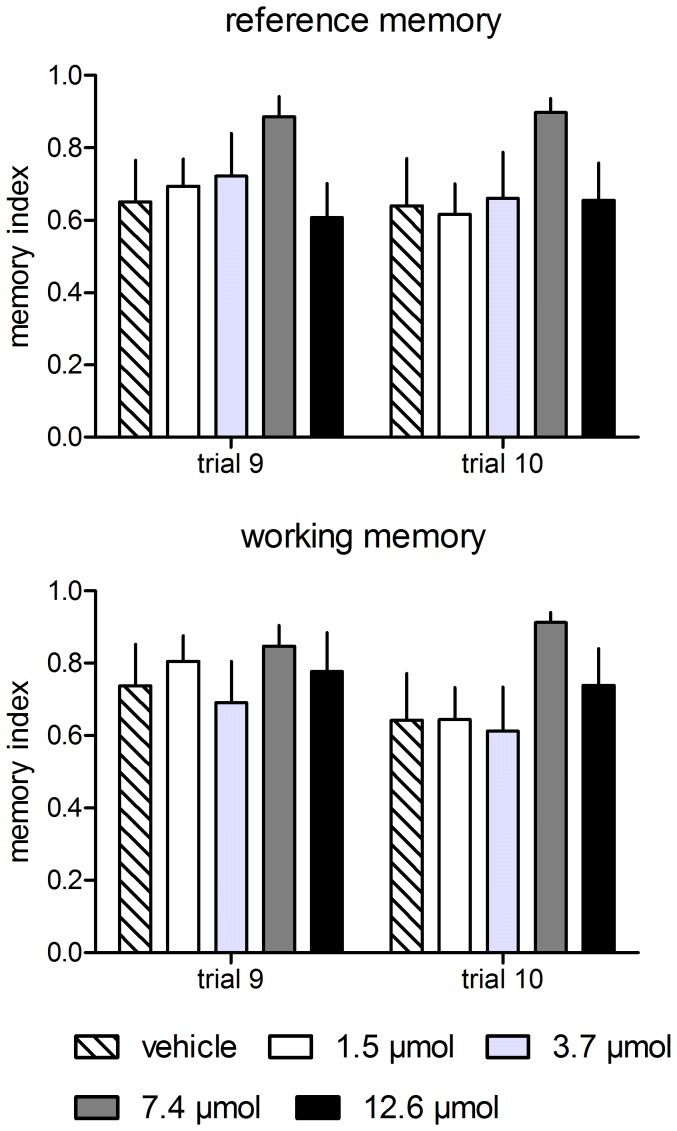
Treatment with MPP at any concentration had no statistically significant effect on the reference and working memory indices at the end of the second acquisition phase (trial 9) and during retention (trial 10). However, animals treated with 7.4 µmol MPP showed the highest indices of both types of memory. Given are the arithmetic means and the s.e.m.

In addition to these behavioral parameters, we analyzed the working and reference memories. For investigating and comparing different memory states, we calculated the error indices for the end of the acquisition (trial 9) and for retention trial 10 (Figure 2). We found a significant overall difference in the reference memory (chi^2^ = 9.75, df = 4, p = 0.045) but not working memory (chi^2^ = 1.19, df = 4, p = 0.880) indices in trial 9. However, single group comparisons revealed no significant differences in reference memory indices between any groups (p>0.1, each). In trial 10, no significant differences in the reference memory (chi^2^ = 8.38, df = 4, p = 0.079) or working memory (chi^2^ = 7.85, df = 4, p = 0.097) indices were identified.

### Hormones

**Figure 3:**
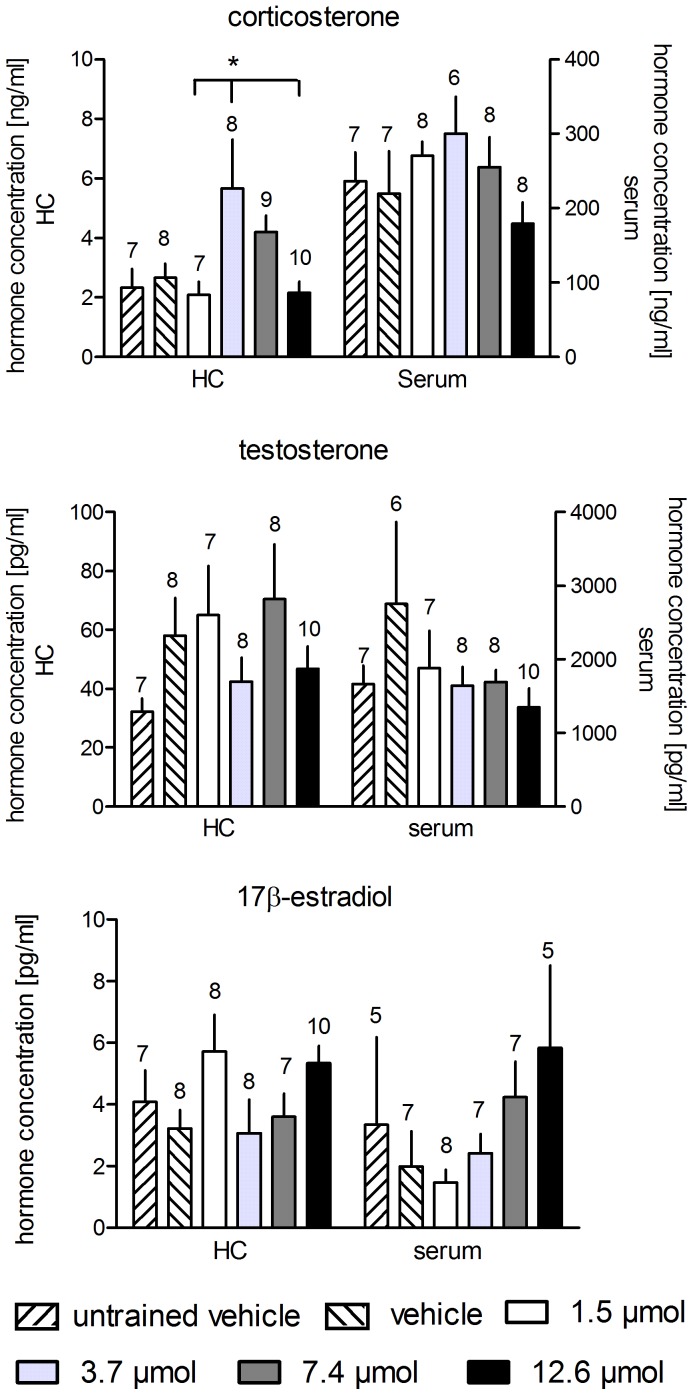
Treatment with MPP at any concentration had a statistically significant effect on the hippocampal concentrations of corticosterone only between the group treated with 3.7 µmol compared to the groups treated with 1.5 µmol and 7.4 µmol MPP. Serum concentrations were not different, and no difference between groups was found for testosterone and 17β-estradiol. Horizontal lines with asterisks indicate statistically significant differences. Numbers above columns indicate sample sizes. Longer vertical lines indicate the groups against which the other groups were tested. Given are the arithmetic means and the s.e.m.

We found significant differences in the hippocampal corticosterone (F_5,48_ = 3.268, p = 0.014) levels, but not in the serum concentrations (F_5,43_ = 1.125, p = 0.364). The statistical analyses of the testosterone and estradiol concentrations (Figure 3) revealed no differences in the hippocampal (F_5,47_ = 1.343, p = 0.265, and F_5,47_ = 1.665, p = 0.164, respectively) and serum samples (F_5,45_ = 1.017, p = 0.420, and F_5,38_ = 1.193, p = 0.334, respectively).

The post hoc tests revealed higher hippocampal corticosterone concentrations in the animals treated with 3.7 µmol MPP compared to the rats treated with 1.5 µmol (p = 0.044) and 12.6 µmol (p = 0.026) MPP.

### Genes

**Figure 4:**
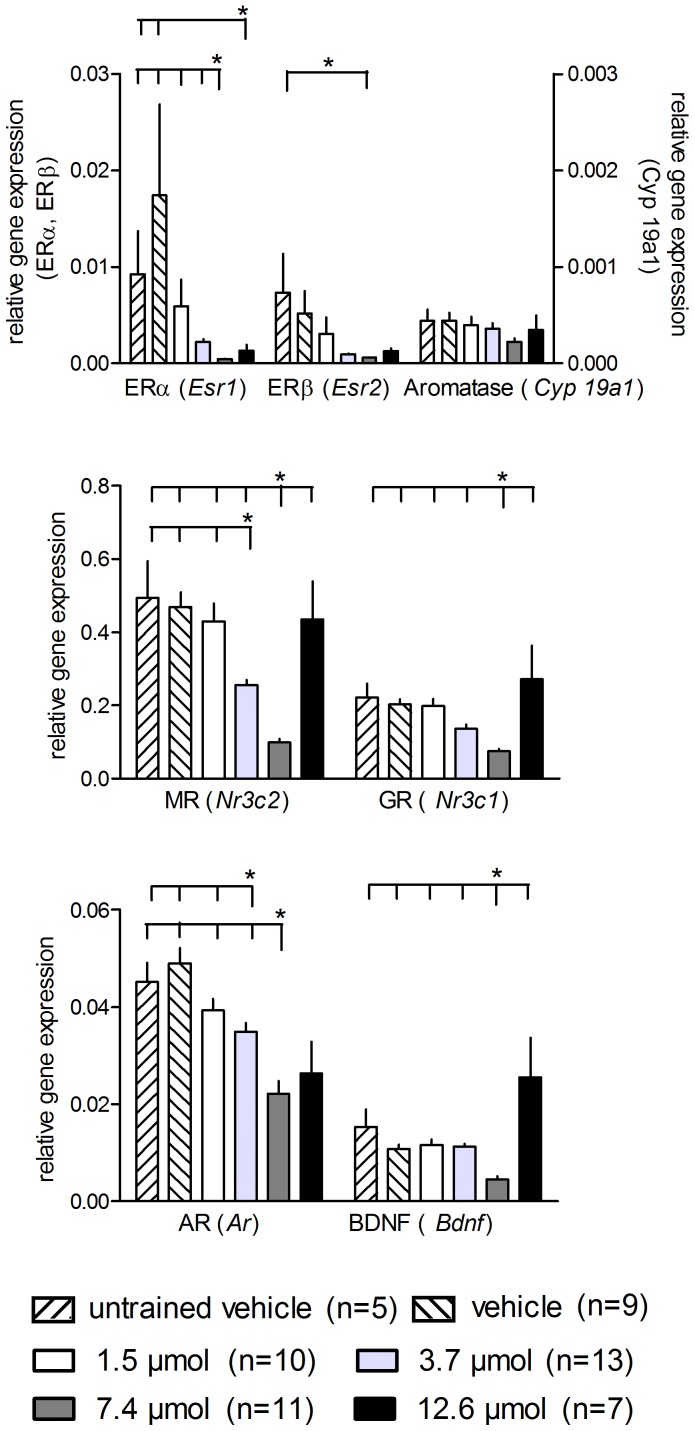
Treatment with MPP had an effect on hippocampal relative gene expression of ERα, MR, GR, AR, and BDNF, but not ERβ and aromatase (*Cyp 19*) encoding genes in a dose-dependent manner with statistically significant lower expression of only the group treated with 7.4 µmol MPP compared to the trained vehicle-treated animals. Horizontal lines with asterisks indicate statistically significant differences. Numbers in brackets indicate sample sizes. Longer vertical lines indicate the groups against which the other groups were tested. Given are the arithmetic means and the s.e.m.

The gene expression data are given in Figure 4. The GLM provides significant differences for all genes (ERα: F_5,54_ = 6.509, p<0.001; ERβ: F_5,54_ = 2.852, p = 0.024; AR: F_5,54_ = 7.567, p<0.001; MR: F_5,54_ = 28.802, p<0.001; GR: F_5,54_ = 11.714, p<0.001; BDNF: F_5,54_ = 11.805, p<0.001) except the aromatase gene *Cyp19* (F_5,54_ = 1.642, p = 1.670).

The single group comparisons revealed that animals treated with 7.4 µmol MPP showed the highest suppression of relative gene expression of ERα compared with animals treated with 1.5 µmol (p = 0.008), 3.7 µmol (p = 0.017), and vehicle (p = 0.004) and untrained vehicle-treated control rats (p = 0.002). Furthermore, animals administered 12.6 µmol MPP had a significantly lower ERα-mRNA level than the vehicle-treated trained (p = 0.036) and untrained (p = 0.014) controls. The relative gene expression of ERβ, however, differed significantly only in the 7.4 µmol MPP-treated rats compared to the untrained vehicle-treated rats (p = 0.043).

The expression pattern of the androgen receptor was similar to that of ERα (7.4 µmol/1.5 µmol: p = 0.011; 7.4 µmol/3.7 µmol: p = 0.042; 7.4 µmol/vehicle: p<0.001; 7.4 µmol/untrained vehicle: p = 0.013; 12.6 µmol/vehicle: p = 0.001; 12.6 µmol/untrained vehicle: p = 0.015). In addition, the 12.6 µmol MPP group showed a difference in AR expression compared with the 1.5 µmol MPP-treated animals (p = 0.017).

The mineralocorticoid receptor also had the lowest gene expression in animals treated with 7.4 µmol MPP compared to all other groups (7.4 µmol/1.5 µmol: p<0.001; 7.4 µmol/3.7 µmol: p<0.001; 7.4 µmol/12.6 µmol: p<0.001; 7.4 µmol/vehicle: p<0.001; 7.4 µmol/untrained vehicle: p<0.001). Moreover, the 3.7 µmol MPP group differed significantly from the 1.5 µmol MPP group (p = 0.030), as well as from the vehicle-treated trained (p = 0.005) and untrained (p = 0.028) control groups.

For the BDNF and glucocorticoid receptor, we observed decreased gene expression in animals treated with 7.4 µmol compared to the 1.5 µmol (both: p<0.001), 3.7 µmol (BDNF: p<0.001; GR: p = 0.005), 12.6 µmol (both: p<0.001), and vehicle-treated trained (both: p<0.001) and untrained (both: p<0.001) groups.

### Dose Effects

**Figure 5:**
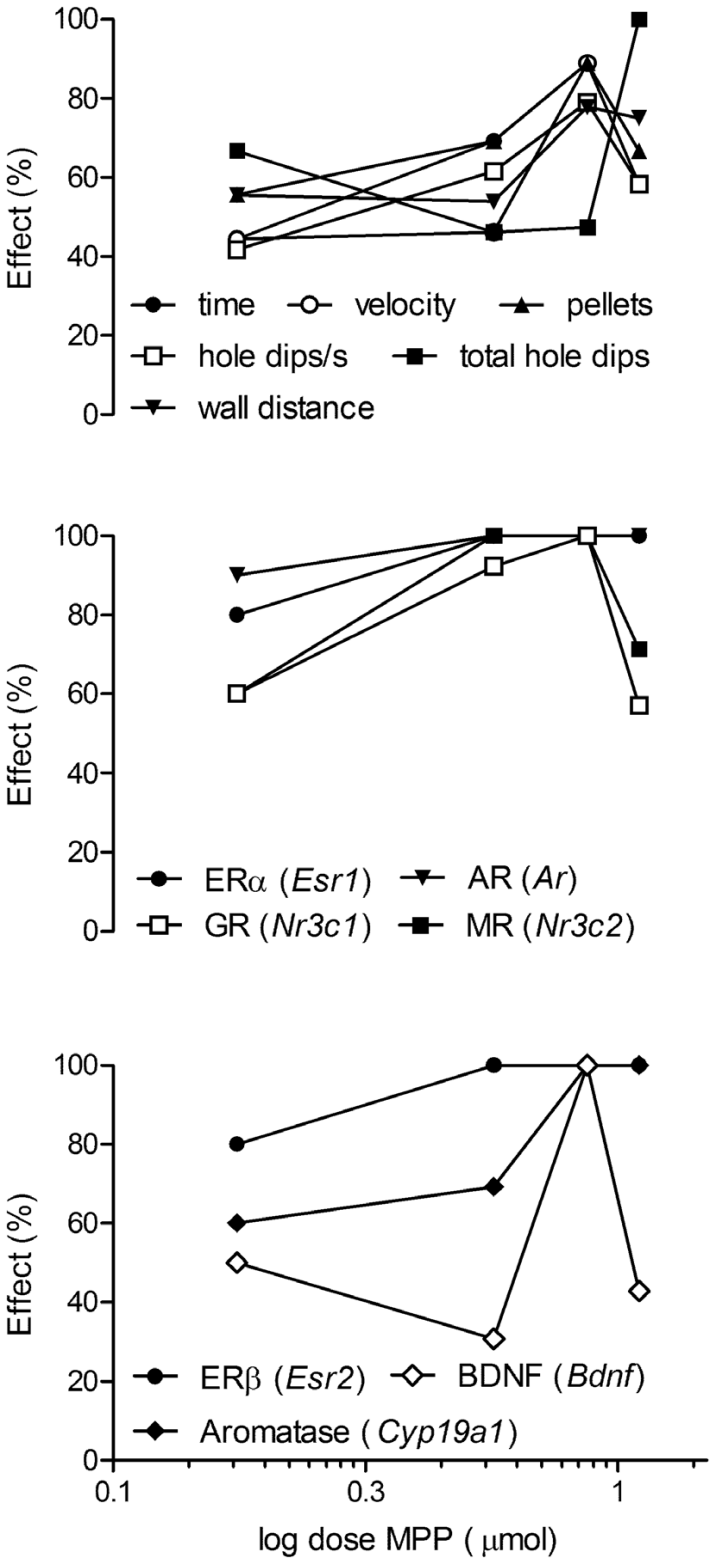
Dose-response relation of behavior (upper panel; time: time to find all pellets, pellets: pellets found) and gene expression for MPP treatment. Effect is given as the percentage of treated animals showing a measurement above (behavior, except time to find all pellets) or below (gene expression, time to find all pellets) the mean for the controls during retention. Corresponding highest effects at dose 7.4 µmol MPP.

To compare dose effects on behavior and gene expression, we calculated a dose-effect curve (Figure 5) at the individual level. We followed the trend indicated by the between-group statistical effects and calculated the curve as the percentage of treated individuals that showed measurements higher (behavior) or lower (gene expression) than the mean of the control animals. The percentage measures were plotted against the logarithm of the dosages. It became immediately apparent that all effects (except total hole dips) were highest at the 7.4 µmol dosage. Thus, only when almost all individuals showed suppression in the expression of all genes was a behavioral effect observed. In addition, ERα, ERβ, and AR gene expression was the most sensitive to the treatment. Effects of more than 50% were observed at the lowest concentration and reached a plateau at 3.7 µmol MPP. Ninety to hundred percent of the individuals responded at 3.7 µmol MPP in MR and GR expression and maintained the effects at 7.4 µmol, whereas the aromatase and BDNF gene expression was less sensitive but also reached 100% responders at 7.4 µmol MPP. Interestingly, with the decay in responders at 12.6 µmol in behavior, the expression of MR, GR, and BDNF increased, whereas ERα, ERβ, and AR expression remained at the 100% level.

### Open-field and Elevated Plus Maze Tests

**Figure 6:**
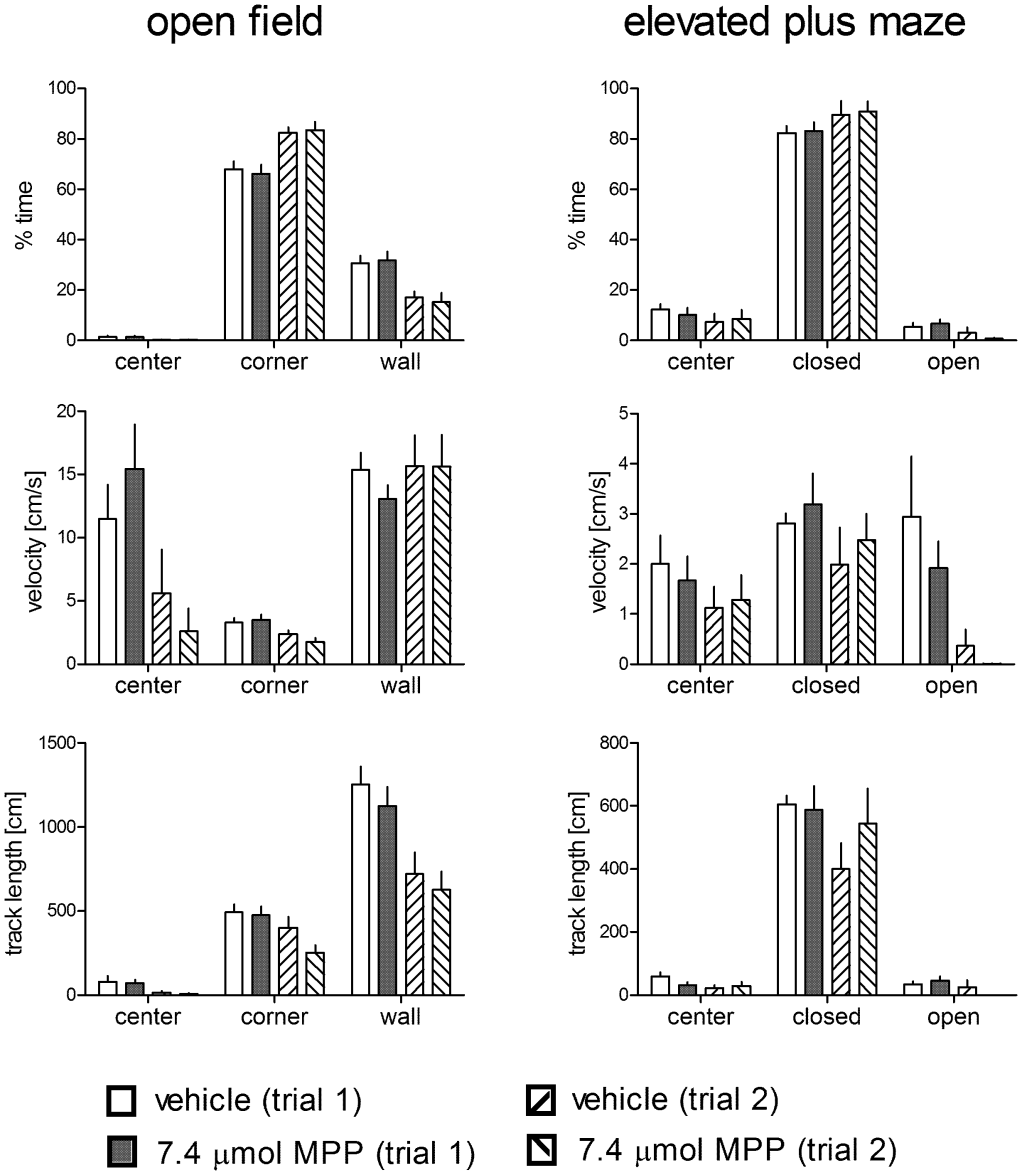
Behavioral results for the open-field (left panel) and the elevated plus maze (right panel) tests of animals treated with 7.4 µmol MPP or vehicle. Closed: closed arms; open: open arms. No group×trial interaction and no group differences were detected for any behavioral parameter. Given are the arithmetic means and the s.e.m.

To test whether the effects observed in the holeboard merely resulted from unspecific effects on locomotor activity or differences in anxiety induced by the pharmacological treatment, separate groups of animals underwent open-field and elevated plus maze tests (Figure 6). These animals were treated with 7.4 µmol MPP (the most effective dose in the holeboard experiments, n = 12, one animal with missing values at day 1) or vehicle (n = 12; due to technical reasons, we lost the data for one animal in the elevated plus maze test at day 2). We found no statistically significant interaction between treatment and trial and no treatment effects in the open field (time spent in the center F_1,22_ = 0.06, p = 0.815; F_1,22_ = 0.01, p = 0.932; time spent in corners F_1,22_ = 0.54, p = 0.471; F_1,22_ = 0.01, p = 0.911; time spent in wall zones F_1,22_ = 0.71, p = 0.407; F_1,22_ = 0.01, p = 0.941; velocity in the center F_1,22_ = 2.58, p = 0.122; F_1,22_ = 0.02, p = 0.894; velocity in the corners F_1,22_ = 1.37, p = 0.254; F_1,22_ = 0.39, p = 0.54; velocity in wall zones F_1,22_ = 0.51, p = 0.483; F_1,22_ = 0.261, p = 0.615; track length in the center F_1,22_ = 0.00, p = 0.995; F_1,22_ = 0.12, p = 0.734; track length in the corners F_1,22_ = 2.10, p = 0.161; F_1,22_ = 1.81, p = 0.192; track length in the wall zones F_1,22_ = 0.04, p = 0.839; F_1,22_ = 0.66, p = 0.425). Similarly, no treatment trial interaction and no treatment effect were determined in the elevated plus maze test (time spent in the center F_1,20_ = 0.54, p = 0.473; F_1,20_ = 0.00, p = 0.956; time spent in closed arms F_1,20_ = 0.00, p = 0.970; F_1,20_ = 0.05, p = 0.82; time spent in open arms F_1,20_ = 2.23, p = 0.15; F_1,20_ = 0.02, p = 0.904; velocity in the center F_1,20_ = 0.18, p = 0.675; F_1,20_ = 0.00, p = 0.987; velocity in closed arms F_1,20_ = 0.01, p = 0.907; F_1,20_ = 0.79, p = 0.383; velocity in open arms F_1,20_ = 0.13, p = 0.720; F_1,20_ = 0.78, p = 0.387; track length in the center F_1,20_ = 2.50, p = 0.129; F_1,20_ = 0.54, p = 0.472; track length in closed arms F_1,20_ = 1.18, p = 0.291; F_1,20_ = 0.61, p = 0.443; track length in open arms F_1,20_ = 2.22, p = 0.152; F_1,20_ = 0.07, p = 0.789).

## Discussion

We found dose-dependent changes (up to 7.4 µmol MPP) in behavioral performance with most significant effects in animals treated with 7.4 µmol MPP. These animals needed less time to find all pellets, moved faster, had more hole dips, and found more pellets than the control animals. In contrast to our hypothesis, the MPP-treated animals performed better regarding motivation (velocity, hole dips/s), whereas, in line with our hypothesis, the cognitive component of the task was not affected by the treatment. Treatment effects on the general locomotor activity and changes in anxiety, which could have contributed to the observed differences, can be ruled out by our open-field and plus maze tests. Regarding exploration and anxiety, the MPP-treated rats did not behave differently from the controls.

The effects of the MPP treatment are particularly surprising, because we found previously that the upregulation of hippocampal ERα gene expression is a learning-related holeboard effect in males at the age tested here. We also found, although not significant, upregulation of ERα gene expression in vehicle-treated males compared to untrained animals. However, the MPP-treated animals showed significantly reduced levels of gene expression not only for ERα but also for MR, GR, AR as well as the BDNF gene, whereas ERβ and aromatase gene expression was unaffected in the trained animals. These results support two conclusions: i. “Specific” steroid receptor functions can be revealed only against the steroid network background, characterized by a stronger relation of ERα activity to its own gene expression and to that of glucocorticoid and testosterone binding androgen receptors than to ERβ, and an effect on the BDNF but not on the aromatase encoding gene. ii. The training-induced increased expression of the ERα encoding gene does not positively correlate with the receptor function, because the receptor blockade results in increased rather than impaired performance of motivation-indicating behavior. Activation of extranuclear ER induces hippocampal BDNF signaling, thus mediating its effects on neuroprotection and plasticity [33], [39]. Because of these known interactions, we included the BDNF gene in the present study, and interestingly, we found the MPP treatment affected BDNF gene expression. Hippocampal BDNF and the related receptors are involved in learning and memory [40], [41]; thus, an ER-BDNF interaction may partly play a role in mediating behavior in the present study.

Thus, at least parts of the observed system of steroid receptors, and there are many others [42], react in a coordinated and dose-dependent manner to the MPP treatment, which suggests that compensating mechanisms exist within the network to keep the system in homeostasis and to provide a mechanism for reacting quickly to external signals. The apparent discrepancy between the previously observed positive correlation of ERα-mRNA with motivational behavior and the failure to reduce motivation by ERα blockade on the protein level in the present study, may be explained by the translation of preexisting, training-induced, silent ERα-mRNA that could be quickly and locally translated into functional receptors [43], [44] by extracellular signaling. MR, that has been identified as the crucial acute stress-related receptor in our holeboard paradigm [45] may be involved in the transduction of external signals. These mechanisms could provide effects upon behavior independently from rapid changes in the availability of locally produced estrogens [23]. Future studies should prove this with in situ-hybridization and optical methods for identifying the hippocampal steroid receptor-mRNA distribution and trafficking in trained and untrained animals. Membrane- and cytosol-specific westernblots can detect training-induced changes in site-specific functional steroid receptors within the hippocampus. The behavioral effect that motivation increased with the ERα-blockade is likely a result of the MPP treatment that reduces ligand binding with functional receptors, which occupation with a natural ligand otherwise leads to a decrease in motivation. Thus, ERα activation does not promote motivational behavior but may constrain over-motivated, high-risk behavior preserving the animal from life-threatening situations. This may be adaptive especially in young post-pubertal rats that ontogenetically are in the phase of migrating from their social groups and exploring new environments. Importantly, we did not find upregulation of ERα gene expression in older individuals in the same situation [24].

The network characterization is further supported by the fact that we found significant effects in behavior only when the expression of all genes (except the aromatase and ERβ encoding genes) was affected, although there were differences in the sensitivity to MPP treatment between genes, as suggested by the dose response curve. Second, changes in hormone concentrations, due to the treatment, were absent. Therefore, compensatory modulations of receptor expression resulting from altered hormonal states cannot explain the results. Last, the effects of MPP on gene expression are dose dependent (up to 7.4 µmol MPP), thus indicating not unspecific treatment-induced downregulation of gene expression. Further, not all genes were affected.

We noted dose-dependent effects of the MPP treatment on all behavioral elements and only an interaction of dose and learning phase effects for the times to find all pellets. Thus, the differences remained stable over the training for most of the behavioral parameters. The partial but not completely similar behavioral results in animals treated with 3.7 and 7.4 µmol MPP (especially for the learning phase×dose interactions) along with the differences in the effects on gene expression between the two dosages suggest complex effects of structural changes in the receptor network on motivation and behavior and may indicate a structural change in the receptor network over training. Training phase–specific changes in correlations of hippocampal steroid hormone concentrations with behavior has been described for the same learning procedure [46]. In that study, hippocampal testosterone concentrations switched from a negative correlation with reference memory errors at acquisition phase 1 to no correlation during acquisition 2 and back to a negative correlation during retention. Corticosterone concentrations in the prefrontal cortex negatively correlated with reference memory errors during acquisition and positively during retention. This may be related to brain region–specific changes in steroid functions in behavioral regulation during training or reflect network adjustments over different brain regions.

Surprisingly, the dose-dependent effects of MPP on behavioral performance disappeared when the animals were treated with a higher dose than 7.4 µmol MPP. Animals treated with 12.6 µmol MPP showed only a larger wall distance when compared to the vehicle-treated group. Notably, this group also showed similar MR and GR expression as the vehicle-treated animals and significantly higher expression of these receptor genes compared to the group treated with 7.4 µmol MPP. Thus, higher concentrations of MPP may result in unspecificity of the receptor blockade with a subsequent rescue of MR and GR expression and behavior.

Due to the intra-cerebroventricular administration of MPP, resulting in brain-wide distribution, other brain regions such as the amygdala and hypothalamus, which contain estrogen receptors [47], [48], [49], are probably involved in the observed behavioral effects. Although no effects of ERα knockdown in the medial amygdala on sexual or aggressive behavior in 16-week-old male mice were observed, the same procedure in the ventromedial nucleus of the hypothalamus reduced both types of behavior [7]. The effects, however, may be age dependent [50], because different interactions and contributions of ERα and ERβ in maintaining hippocampal-dependent memory during aging in female mice have been reported [51]. Future studies, including local drug administration, should reveal the specific contributions of these brain regions to the motivational states and behavioral performance in young rats and the possible rules underlying the experience-dependent structural changes in the steroid network.

## Supporting Information

Video S1 Representative example of the behavioral performance of a rat treated with 7.4 µmol MPP (trial 10).
(MPG)

Video S2 Representative example of the behavioral performance of a rat treated with vehicle (trial 10).
(MPG)
